# Enhanced chiral discrimination in mass spectrometry with orbital angular momentum beams

**DOI:** 10.1126/sciadv.aec6549

**Published:** 2026-06-05

**Authors:** Haritha Venugopal, Parishkrith Aravind, Angitha Sajeevan, Sanket Sen, Abhisek Sinha, Sucharita Giri, Gopal Dixit, Ram Gopal, Vandana Sharma

**Affiliations:** ^1^ Department of Physics, Indian Institute of Technology Hyderabad, Kandi, Sangareddy 502285, India.; ^2^ Synchrotron SOLEIL, L'Orme des Merisiers, Départementale 128, 91190 Saint Aubin, France.; ^3^ Department of Physics, Indian Institute of Technology Bombay, Powai, Mumbai 400076, India.; ^4^ Max-Born Institute, Max-Born Straße 2A, 12489 Berlin, Germany.; ^5^ Tata Institute of Fundamental Research Hyderabad, Serilingampally, Hyderabad 500046, India.

## Abstract

Chirality, the absence of mirror symmetry, is a fundamental property observed from subatomic particles to molecules and macroscopic systems. Distinguishing molecular mirror images (enantiomers) is essential in pharmaceuticals and molecular diagnostics. Traditional chiral spectroscopy relies on chiral light carrying spin angular momentum (SAM). Here, we demonstrate that twisted femtosecond laser beams carrying orbital angular momentum (OAM), combined with SAM and mass spectrometry, can enhance chiral selectivity by up to fourfold. Using the enantiomers (1S)-(-)- and (1R)-(+)-Camphor, we observe strong enantioselective ionization and fragmentation with laser pulses of a few hundred femtoseconds and energies of several hundred μJ. The interplay of SAM and OAM improves the sensitivity and selectivity of chiral mass spectrometry without chemical derivatization, offering a scalable approach for probing molecular handedness in complex chemical and biological systems.

## Teaser

Structured light carrying spin and orbital angular momentum induces a mass-selective chiral response.

## INTRODUCTION

Chirality is a fundamental geometric property that manifests across all scales of matter, from molecular to macroscopic, playing a critical role in determining chemical functionality and biological processes. Chiral molecules exist in two nonsuperimposable mirror-image forms, called enantiomers, which can exhibit markedly different interactions with other chiral systems, including biological targets (*1*). As a result, chirality is not only a fundamental asymmetry (*2*, *3*) but is also a critical factor in applications such as drug development (*4*, *5*) and enantioselective catalysis (*6*), among others. Established approaches to enantiomer discrimination include chiral high-performance liquid chromatography (*7*), which relies on chemical separation using chiral stationary phases or derivatization and therefore requires extensive sample preparation. Spectroscopic approaches to chiral analysis rely on the interaction between chiral molecules and polarized light. Optical rotation measures the rotation of plane-polarized light, while advanced methods such as circular dichroism (CD), vibrational CD (VCD) (*8*), and Raman optical activity (ROA) (*9*) detect the differential scattering of circularly polarized light. These methods typically yield small asymmetries in absorption or scattering, often requiring high sample concentrations and complex optical setups. VCD signals are often weaker than electronic CD by orders of magnitude, while ROA requires high analyte concentrations and extended acquisition times (*9*). More recently, frequency comb–based optical spectroscopy has enabled highly sensitive chiral measurements, with sophisticated instrumentation (*10*). An alternative approach uses photoionization, particularly photoelectron CD (PECD) (*11*), where circularly polarized light ionizes randomly oriented chiral molecules, and the angular distribution of the emitted photoelectrons exhibits forward-backward asymmetry. PECD relies solely on electric dipole interactions and offers a good enantiomeric contrast of 10% with higher sensitivity than conventional methods. Photoion yields from left and right circularly polarized light [photo-ion CD (PICD)] also exhibit enantioselectivity and have been studied extensively (*12*–*17*), with differences typically of a few percent. Advancing simple yet robust approaches to enantiomeric discrimination that deliver high chiral contrast would thus enable broader possibilities across pharmaceutical and analytical applications.

Here, we introduce a method that combines structured chiral light-induced ionization with mass spectrometric detection of fragment ions to distinguish enantiomers. We demonstrate that our approach eliminates the need for derivatization or elaborate sample preparation, operates in the gas phase, and does not rely on angle-resolved electron or ion detection. We find that the optimal conditions for signal enhancement can be achieved by tailoring the pulse duration and intensity of the structured light. The underlying mechanism of our approach is based on the interference between electric dipole and electric quadrupole interactions, which markedly enhances the chiral sensitivity. This stands in contrast to other techniques that rely on electric-magnetic dipole interactions for chiral discrimination (*18*–*20*). Beyond fundamental insights, this work provides a structured light-enabled framework for chiral analysis, with potential relevance for ultrafast spectroscopic techniques and chemical sensing.

**Fig. 1.**
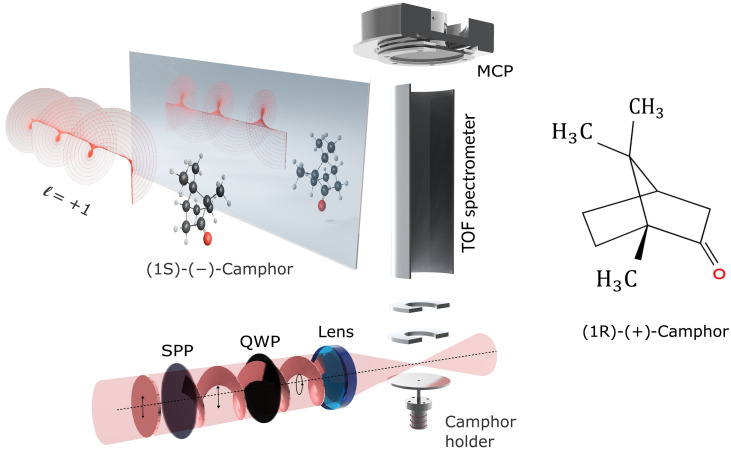
Schematic illustration of the interaction between (1S)-(−)- or (1R)-(+)-Camphor molecule and structured chiral light, followed by mass spectrometric detection of fragment ions. In the top-left section, the cartoon depicts phase fronts of a light beam containing OAM, ℓ=+1 and its mirror image with ℓ=−1 interacting with (1S)-(−)-Camphor and (1R)-(+)-Camphor, respectively. The azimuthal variation of the phase (radially equidistant curves) leads to a helical structure of the wavefront. A spiral phase plate (SPP) imposes a topological charge of ℓ=±1,±2 to the beam of femtosecond laser pulses (bottom, red). The incident linear polarization is converted to circular polarization using a quarter-wave plate (QWP). A lens focuses the pulses to intensities of the order of 10^12^ to 10^13^ W/cm^2^, which is sufficient to trigger multiphoton ionization and subsequent fragmentation. Fragmented ions are collected and dispersed in time to record the time-of-flight (TOF) spectrum using a microchannel plate (MCP) detector and anode. The top-right shows the molecular structure of (1R)-(+)-Camphor.

Fundamentally, left- and right-circularly polarized light carry opposite spin angular momentum (SAM) along the propagation direction ($J_{z}=\mathrm{s\hbar}=\pm \hbar$) corresponding to the two possible handedness states of circular polarization. Recent advances in light structuring have introduced orbital angular momentum (OAM) as an additional degree of freedom, distinct from SAM. Light carrying OAM exhibits helical phase fronts characterized by a topological charge ℓ, and each photon carries an angular momentum of charge ±ℓℏ (*21*). The sign of ℓ determines the handedness of the wavefront, giving rise to its chiral or twisted character that originates purely from its spatial phase profile, distinct from its polarization state. In Fig. 1, the top-left section contains a depiction of a light beam with topological charge ℓ=+1 and its mirror image ℓ=−1 showing the twisted nature of the phase front. Interactions between OAM beams and matter have shown promise in optical trapping (*22*), microscopy (*23*), quantum optics (*24*), attosecond chronoscopy (*25*), and conical intersections (*26*). In chiral discrimination, helical dichroism—the differential response to beams of opposite OAM charges—has been observed in engineered nanostructures (*27*) and disordered media (*28*).

In the following work, we irradiated Camphor enantiomers with 850-fs pulses at intensities ranging from 10^12^ to 10^13^ W/cm^2^ using structured beams of topological charges ℓ=±1,±2, with s=±1. We unequivocally demonstrate that the fragment ion yield exhibits a clear dependence on the sign of ℓ with opposite enantiomers producing reversed asymmetries. This ionization sensitivity to both OAM and SAM constitutes a hitherto unexplored pathway for optical chiral recognition in a nonlinear regime, which holds significance given recent predictions of two-photon activity (*29*). Recent studies have also highlighted the chiral sensitivity enabled by structured light (*30*, *31*). However, the chiral sensitivities are comparable to those reported for PICD. Thus, while the state of the art in enantiomer discrimination continues to evolve, mass spectrometry–based approaches such as the one demonstrated here represent an important step forward, illustrating one of the first experimental implementations of chiral discrimination directly in mass spectra using structured light.

## RESULTS

**Fig. 2.**
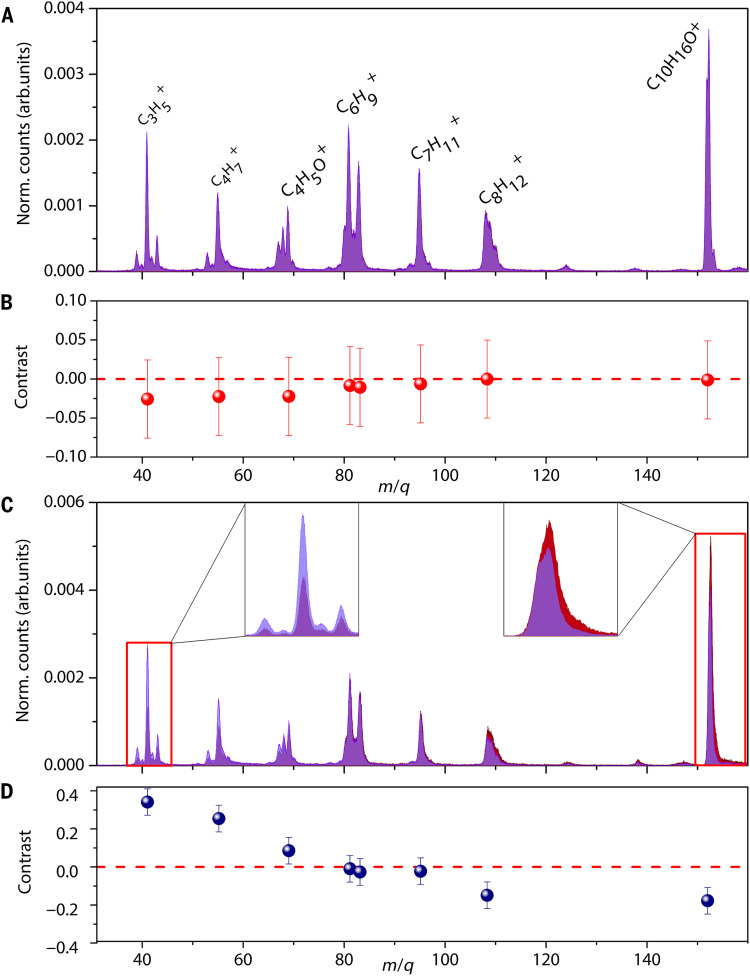
Mass spectra of fragment ions and contrast for SAM alone and when combination of OAM and SAM of the laser pulse is used, recorded for (1S)-(−)-Camphor. (**A**) Mass spectra, normalized ion counts as function of m/q of (1S)-(−)-Camphor with (ℓ=0,s=+1) in blue and $\left(\ell^{\prime }=0,s^{\prime }=-1\right)$ in red (only SAM), which nearly superimpose on each other, leading to a low contrast (**B**) as shown in the ion fragments of (1S)-(−)-Camphor. (**C**) Mass spectra of (1S)-(−)-Camphor with (ℓ=+1,s=+1) in blue and ($\ell^{\prime }=-1,s^{\prime }=+1$) in red (OAM and SAM). The insets in (**C**) show magnified views of the mass spectra around m/q=41 and m/q=152. (**D**) Corresponding contrast for the mass spectra shown in (C). The laser pulse is 850 fs long with an intensity of 2.2 × 10^12^ W/cm^2^.

A schematic of our experimental setup is shown in Fig. 1, where femtosecond laser pulses with OAM and/or SAM interact with an effusive gas jet containing an enantiopure ensemble of Camphor molecules, resulting in the ionization and fragmentation of the molecules. We obtain the mass spectrum m/q from time-of-flight measurements of the ionic fragments, and we normalize all spectra presented here to the total ion counts of their respective datasets. Figure 2A displays the mass spectrum of (1S)-(−)-Camphor under the influence of SAM alone (ℓ=0) for a laser pulse of 850 fs with an intensity of 1.2 × 10^12^ W/cm^2^. The red-shaded spectrum (s=+1) closely overlaps with the blue-shaded spectrum $\left(s^{\prime }=-1\right)$, indicating a poor contrast in CD. We acquired the spectra for different polarization states of the laser for the same amount of time (see the “Experimental design” section in Materials and Methods).

Camphor, with its highest occupied molecular orbital primarily attributed to the lone pair located on the carbonyl oxygen, has an ionization energy of ~8.7 eV. The appearance energy for fragment ions is reported as ~9.7 eV in single-photon ionization experiments (*32*). The fragment ion ${C_{8}H_{12}}^{+}\;\left(m/q=108\right)$ arises from the loss of one of its rings containing the carbonyl group (*33*). Similarly, ${C_{7}H_{11}}^{+}$ is formed via simultaneous losses of the ketene and methyl groups. The unraveling of both cyclic structures, accompanied by the loss of neutral CO and other hydrocarbon groups, results in linear fragments with (m/q=81,83), respectively. The $C_{4}H_{5}O^{+}\;\left(m/q=69\right)$ fragment is accompanied by a cyclic hydrocarbon neutral, whereas ${C_{4}H_{7}}^{+}\;\left(m/q=55\right)$ and ${C_{3}H_{5}}^{+}\;\left(m/q=41\right)$ are the ionic counterparts of this cyclic hydrocarbon. Figure 2B presents the contrast between the yields for the two polarization states for each fragment ion. To quantify chiral sensitivity, we define the contrast G as the ratio of the difference to the sum of total ion fragment yields for the two spin and/or OAM states of light as follows

$$G=\frac{N_{\ell ,s}-N_{\ell^{\prime },s^{\prime }}}{N_{\ell ,s}+N_{\ell^{\prime },s^{\prime }}}$$ (1)

To elucidate the role of SAM and OAM in chiral recognition, we conducted a series of systematic experiments using all possible combinations of SAM and OAM values of the laser pulses as presented in Table 1. The contrast G in the total ion counts between ℓ=0,s=1 and $\ell^{\prime }=0,s^{\prime }=-1$ is found to be 0.07, with similar values for the fragment ions. For comparison, PICD values for chiral molecules are experimentally found to be 1 to 10% (*34*). G for the achiral reference molecule O_2_ was 0.05 (SAM alone), defining the lower limit of detectable CD signals in this study. Figure 2 (C and D) displays the mass spectra and the contrast of (1S)-(−)-Camphor under the influence of laser pulses carrying both OAM and SAM. The spectra were recorded at a pulse width of 850 fs with an intensity of 2.2 × 10^12^ W/cm^2^. The total ion contrast for different combinations of (ℓ,s) pairs is listed in Table 1. Figure S1 illustrates the dependence of *G* on ellipticity.

**Table 1.** Contrast for (1S)-(−)-Camphor [rows (a) to (e)] for different pairs of orbital and SAM (ℓ,s). The row (f) presents the measurements for (1R)-(+)-Camphor.

Label	(ℓ,s) pairs	Contrast G
a	(+1, +1); (−1, +1)	0.4
b	(+1, -1); (−1, -1)	0.4
c	(+1, 0); (−1, 0)	0.1
d	(+1, +1); (+1, -1)	0.03
e	(0, +1); (0, -1)	0.07
f	(+2, +1); (−2, +1)	0.31

**Fig. 3.**
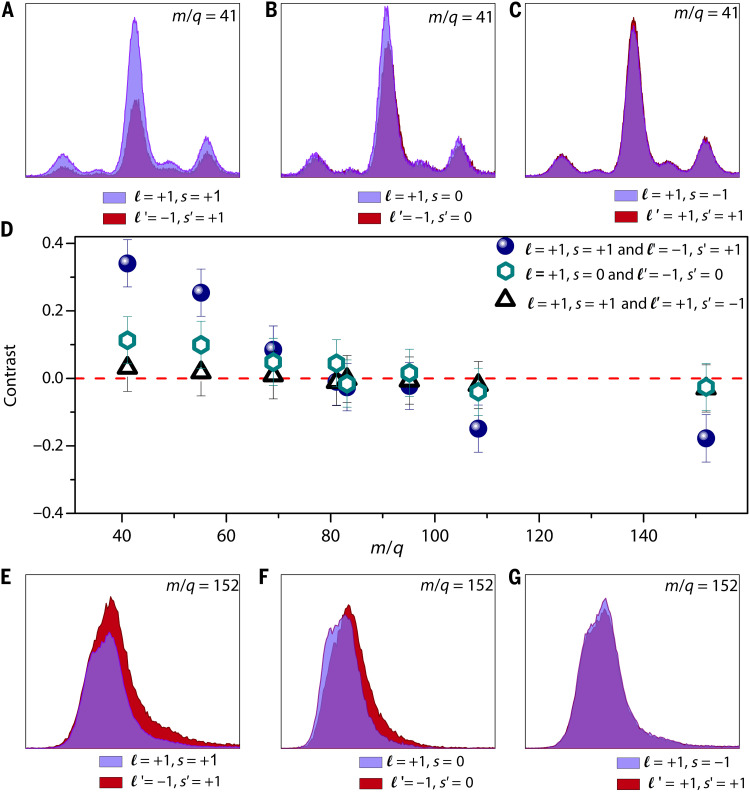
Contrast values for various combinations of OAM and SAM and mass spectra for selected laser-driven fragment ions of (1S)-(−)-Camphor. (**A** to **C**) Magnified views of the mass spectra at m/q=41. (**D**) Contrast for: (ℓ=+1,s=+1) and $\left(\ell^{\prime }=-1,s^{\prime }=+1\right)$, (ℓ=+1,s=0) and $\left(\ell^{\prime }=-1,s^{\prime }=0\right)$, and (ℓ=+1,s=+1) and $\left(\ell^{\prime }=+1,s^{\prime }=-1\right)$. (**E** to **G**) Magnified views of the mass spectra at m/q=152 for different combinations of OAM and SAM values.

A key insight into the role of structured light in enantiosensitive fragmentation ionization emerges from a comparative analysis of different combinations of (ℓ,s) summarized in Table 1 and plotted in Fig. 3. The middle panel (Fig. 3D) in the figure displays the value of G for various combinations of OAM and SAM; the top panel shows a magnified view of the mass spectra around m/q=41. For example, in Fig. 3A, the mass spectrum for (ℓ=+1,s=+1) in blue is overlaid with the mass spectrum for $\left(\ell^{\prime }=-1,s^{\prime }=+1\right)$ in red. Figure 3B displays an overlay of the mass spectrum for (ℓ=+1,s=0) in blue and $\left(\ell^{\prime }=-1,s^{\prime }=0\right)$ in red. In the Fig. 3C, the mass spectrum for (ℓ=+1,s=−1) in blue is overlaid with the mass spectrum for $\left(\ell^{\prime }=+1,s^{\prime }=+1\right)$ in red. Similarly, in the Fig. 3 (E to G), a magnified view of the mass spectra around m/q=152 is presented.

As is evident from the middle panel of Fig. 3D, the value of G remained as low as 0.1 when light carrying only OAM interacted with (1S)-(−)-Camphor, highlighting that OAM alone is ineffective in driving strong chiral discrimination. For reference, we observed a maximum contrast of 0.07 in total ion yields for the achiral O_2_ molecule (with OAM and SAM). Similarly, the combination of identical OAM (ℓ=+1) but opposite SAM (s=±1) yielded a negligible contrast of 0.03 (row d, Table 1), confirming that spin alone, without orbital structure, has limited influence in chiral recognition. The breakthrough arises when OAM is combined with finite SAM, leading to a substantial increase in G to 0.4, as shown in rows a and b in Table 1. This fourfold enhancement in G indicates a synergistic effect, where the spatial helical phase of the light enhances the enantiosensitive response initiated by SAM-driven CD. The marked enhancement in G arises from the interplay between the electric dipole and electric quadrupole interactions as illustrated in the “Theory” section in Materials and Methods. It is important to emphasize that the laser pulse intensity lies in the range of 10^12^ to 10^13^ W/cm^2^, which induces preferential alignment of the randomly oriented Camphor molecules in the interaction region. This induced alignment results in appreciable electric dipole and electric quadrupole interactions, even after averaging the measured signal, as applicable to the present scenario.

**Fig. 4.**
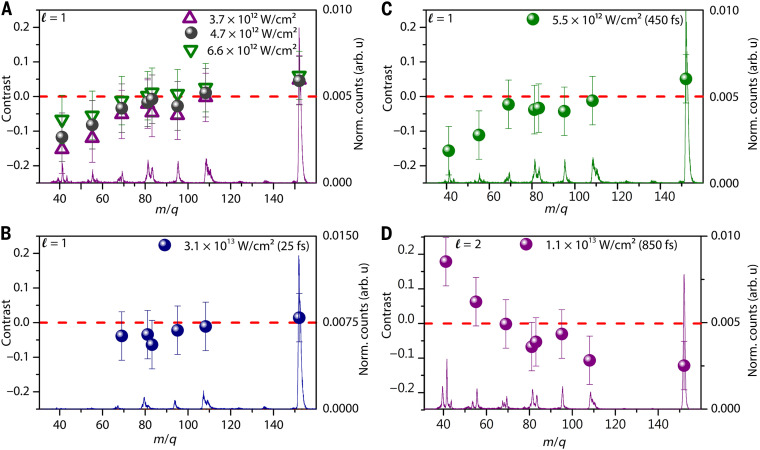
Sensitivity of the mass spectra and contrast of (1R)-(+)-Camphor to different laser parameters and higher-order OAM beams. The mass spectrum (ℓ=+1,s=+1; 3.7 × 10^12^ W/cm^2^) and contrasts for (ℓ=+1,s=+1) and ($\ell^{\prime }=-1,s^{\prime }=+1$) with (**A**) different intensities for the same pulse duration (850 fs), (**B**) pulse width 25 fs with 3.1 × 10^13^ W/cm^2^, and (**C**) pulse width 450 fs with 5.5 × 10^12^ W/cm^2^. (**D**) Mass spectrum (ℓ=+2,s=+1) and corresponding contrast for the combination (ℓ=+2,s=+1) and $\left(\ell^{\prime }=-2,s^{\prime }=+1\right)$ at 850 fs, 1.1 × 10^13^ W/cm^2^.

The fragment ions for (1S)-(−)-Camphor exhibit another notable behavior: Ions with m/q≤(>) 81 have G≥(<) 0 as is evident from Fig. 3D. The sign reversal of G between fragment and parent ions, with an inflection point for m/q=81,83, reflects earlier observations of mass-dependent sign reversal in CD (*35*). Following measurements on (1S)-(−)-Camphor, we conducted experiments on (1R)-(+)-Camphor to assess the sensitivity of our method to discern the handedness. As shown in Fig. 4 (A and C), the total ion contrast was found to invert, producing a value of G = −0.14 with enhanced ion yields observed for $\ell^{\prime }=-1$ relative to ℓ=+1. In addition to the sign reversal in total ion contrast, a corresponding sign flip in G is also observed for individual fragment ions. This inversion of the sign of G provides compelling evidence of the enantiospecificity of our method and highlights its potential to reliably discern molecular chirality.

Until now, our discussion was focused on results obtained using 850-fs laser pulses at an intensity of 2.2 × 10^12^ W/cm^2^. To establish the robustness of our approach, it is essential to investigate how G responds to varying laser parameters. To this end, we performed a systematic series of measurements on (1R)-(+)-Camphor, varying both the pulse duration and the laser intensity. The mass spectra and corresponding contrast between fragment ions under different intensities, for combined OAM and SAM at 850 fs, are shown in Fig. 4A, while Fig. 4C shows the measurement for 5.5 × 10^12^ W/cm^2^ at 450 fs. We systematically varied the laser intensities from 3.7 × 10^12^ to 6.6 × 10^12^ W/cm^2^ to examine its effect on the contrast values. With increased intensities, a reduction in contrast is evident, possibly stemming from saturation in optical transitions. A similar sensitivity of G to laser parameters is also observed for (1S)-(−)-Camphor.

To evaluate the role of pulse duration, we repeated the experiment at the shortest available pulse width of 25 fs at 3.1 × 10^13^ W/cm^2^. Note that we chose the intensities corresponding to each pulse duration to yield similar total ion count rates for this comparison. The resulting mass spectra in Fig. 4C display a reduced degree of fragmentation compared to the 850 fs case. The total ion contrast under these conditions was −0.08, approaching the experimental error margin. The contrast G for total ion yields was found to increase with pulse width and subsequently saturate (fig. S2). A similar trend, wherein the CD signal rises from 50 to 200 fs and plateaus thereafter, has been previously documented (*36*). Our observations align with these findings, reinforcing the significance of pulse width as a tunable parameter for optimizing chiral signal in ultrafast experiments.

We performed additional measurements using OAM states with topological charge ℓ=±2 to examine the contribution of higher-order light-matter interactions. These experiments probed (1R)-(+)-Camphor at longer pulse durations (850 fs), where the chiral contrast is well developed. The measurements were conducted using the SAM-OAM combination (ℓ=+2,s=+1) and $\left(\ell^{\prime }=-2,s^{\prime }=+1\right)$, with a peak laser intensity of 1.1 × 10^13^ W/cm^2^, chosen to match the total count rates obtained in the earlier experiments with ∣ℓ∣=1. Under these conditions, the contrast extracted from the total ion yield was 0.31. The mass spectra and the corresponding contrast values for the individual fragment ions are overlaid and are presented together in Fig. 4D. We observe a clear dependence on the OAM charge. For ℓ=±1, the ion yield is higher for the ℓ=−1 state, whereas for ℓ=±2, the trend is reversed and higher ion counts are obtained for ℓ=+2. This inversion of the yield asymmetry with change in OAM points to the role the parity of the OAM beam plays in the eventual asymmetry in fragmentation. While an increase in contrast is also observed, we have to be careful in the interpretation. We have chosen a single-pulse duration and intensity for the experiments with ∣ℓ∣=2. We note that the radius of maximum intensity $r_{\text{max}}$ varies as $\sqrt{|\ell |}$ for the same focusing geometry (*37*). Theory predicts that the contrast due to OAM effect falls off as ∣ℓ∣r, and the increase in contrast due to ℓ could potentially be counterbalanced. The parity-driven $\left(-1^{|\ell |}\right)$ contrast reversal between ℓ=±1 and ℓ=±2 on the other hand, provides compelling evidence that OAM plays a decisive role in chiral discrimination.

## DISCUSSION

We use a theoretical treatment, grounded in symmetry considerations (see the “Theory” section in Materials and Methods), to provide a qualitative insight into the key results presented here, arising from the combination of SAM and OAM. Spin-orbit coupling between SAM and OAM is known to become pronounced in nonparaxial fields, particularly under tight focusing with high numerical aperture optics (*38*). In the present work, the beam is weakly focused using a low numerical aperture (0.04). Corresponding to the weakly focused paraxial regimes, OAM enters into light-matter coupling through transverse field gradients rather than via strongly nonparaxial longitudinal field components. The OAM-dependent term in our formulation arises from the transverse azimuthal phase gradient of the Laguerre-Gaussian field, ∂/∂ϕ. This contribution is explicitly contained in the ∇E term in Eq. 2 and in its simplified form in Eq. 3. The Power-Zienau-Woolley Hamiltonian include both E1 (dipolar) and E2 (quadrupolar) terms. For a Laguerre-Gaussian beam, the first OAM-dependent contribution enters through the spatial gradients of the field in the quadrupole term, while the pure dipole term is independent of the OAM. The observable contrast then arises from the interference between E1 and E2 amplitudes. Symmetry constraints ensure that the signal corresponding to differential absorption of different signs of ℓ vanishes for linear polarization. Thus, OAM chiral sensitivity is only shown in the presence of nonzero SAM. In Table 1, the value reported for the linearly polarized beam is 0.10, while the corresponding error bar is 0.07. We also note that the linear polarization of the beam, from the Stokes S_1_ parameter, is ~92% (table S1). Polarization impurity may additionally contribute to the small residual contrast observed in the experiment. To further elucidate the role of polarization, we performed measurements for left- and right-circularly polarized beams, linear polarization, and two intermediate ellipticities on (1R)-(+)-Camphor at a pulse duration of 850 fs. The resulting data (fig. S1) show a clear dependence of the measured contrast on the degree of ellipticity, consistent with the expected evolution of the spin contribution in the presence of OAM. Thus, within experimental constraints, a good consistency is seen between our results and the theoretical predictions.

However, mirror-image systems often invite expectations of numerical symmetry. Here, our measurements are the outcome of a highly nonlinear process that depends on several experimental parameters beyond molecular handedness. As a result, the exact numerical values of G for opposite enantiomers, while expected to be equal and opposite in general, are not shown here in our experiments. We have 0.4 for (1S)-(−)-Camphor and −0.14 for (1R)-(+)-Camphor. Repeated experiments reveal a consistent chirality-dependent response: Switching the enantiomer reverses the sign of the effect and preserves its overall qualitative behavior, which confirms that the signal originates from molecular handedness rather than uncontrolled artifacts. The contrast reversals for ℓ=±2 also give credence to an essential symmetry argument, even while being guarded about the actual G values. These observations are not uncommon in experimental investigations of nonlinear chiral processes (*16*, *27*).

We also note that for a strictly isotropic, weak-field ensemble, the E1-E2 average can vanish. Our measurements are performed at high intensities of 10^12^ to 10^13^ W/cm^2^ and pulse durations up to 850 fs, where the anisotropic polarizability of camphor leads to intense laser-induced preferential alignment during the pulse (*39*), breaking exact isotropy and allowing a nonzero orientationally averaged E1-E2 contribution. We observe that for the shortest pulse duration (25 fs), the measured contrast is −0.08, which is very close to the experimental error bar, indicating negligible selectivity. As the pulse duration is increased, the contrast rises and reaching a maximum value at ~450 fs. For longer pulse durations, including 850 fs, the contrast does not increase further and instead remains within the same range, indicating a saturation. For very short laser pulses, the high peak intensity together with the broad spectral bandwidth leads to substantial coupling to multiple excited states. The presence of these additional excitation pathways can reduce the population difference, resulting in a suppression of the chiral contrast. With increasing pulse duration, the excitation becomes more selective. Under these conditions, the population asymmetry increases, leading to an enhanced chiral signal. Furthermore, for laser pulses in the subpicosecond domain, the impulsive alignment is substantial, and even in a room temperature ensemble, the alignment onto the plane of the polarization is observed. The degree of alignment typically increases with the fluence (*40*), which is the case here, as we keep the intensity nearly the same, increasing the pulse duration.

Figure 4A demonstrates that the contrast G exhibits a pronounced intensity dependence, decreasing as the excitation intensity is increased. The trend points to even weak transitions become saturated at higher intensities, causing small differences in absorption or ion yield to vanish, seen commonly in CD measurements (*41*). Strong-field ionization with OAM beams is a consequence of the interplay between peak field strength and field gradients. The field gradients are responsible for helicity dependent ionization component, whereas the peak field strength determines the overall ionization rate. As the laser intensity approaches saturation, the contribution of the peak field strength dominates over that of the field gradients, leading to an enhanced ionization rate but a reduced difference in ionization between the left- and right-handed helical light due to ground state depletion. At intensities well below saturation, the field gradient contribution dominates, leading to enhanced helicity-dependent ionization rate. Extensive numerical simulations will be able to confirm these predictions.

Recent pioneering experiments using OAM beams (*30*, *31*) have demonstrated that the helicity encoded in the optical wavefront can itself act as a chiral probe, providing direct evidence that OAM can couple to molecular handedness and extend chiroptical detection beyond conventional SAM-based techniques. However, the resulting asymmetries remain on the same levels as in PICD measurements. Particularly, notable contrasts were seen only with beams misaligned with respect to the axis of the spiral phase plate. The effect of SAM, as expected from theory or a flip in parity due to higher-order OAM beams following symmetry arguments, is not shown either. Conversely, our results establish the cooperative action of SAM and OAM and the central role of parity in chiral discrimination.

Chiroptical techniques such as VCD, ROA, and PECD have been instrumental in studying molecular chirality. However, each presents specific limitations in sensitivity, sample requirements, and practical applicability. VCD signals in the infrared regime are typically more than two orders of magnitude lower than those of electronic CD in the ultraviolet-visible regime, necessitating high sample concentrations and extended acquisition times (*42*). Similarly, ROA often requires substantial analyte enrichment, with sensitivity limits around 0.2 mM, and prolonged signal acquisition times, which can be impractical for clinical measurements (*43*). Alternatively, PECD and its elliptical counterpart, photoelectron elliptical dichroism (*39*), exhibit a pronounced chiral response, and recent developments combining PECD with standard high-resolution nanosecond laser sources have enabled selective excitation in molecular mixtures and even conformer-specific studies (*44*, *45*). However, the extraction of the forward-backward asymmetry requires coincident ion-electron detection and angularly resolved photoelectron measurements, which can limit their practicality for routine chiral detection measurements.

The results presented here, wherein the combination of SAM and OAM enhances the chiral signal in mass spectrometry, with the highest contrast observed at longer pulse widths, open emerging frontiers for sensitive chiral detection. When the charge on OAM was increased to ℓ=±2, the ion counts were higher for ℓ=+2, reversing the trend observed for ℓ=±1. This behavior directly reflects the influence of the parity on the chiral ionization dynamics. By systematically tuning the pulse duration and intensity, we demonstrate control over the enantioselective ion yield contrast. The distinct and consistent chiral response highlights a robust capability to distinguish enantiomers with enhanced sensitivity. Our approach delivers enhanced sensitivity without the need for large sample quantities. Unlike traditional chemical-based chiral detection methods (*7*, *46*, *47*), our approach eliminates the need for sample preparation or chemical derivatization, thereby offering a potential practical advantage over existing techniques.

## MATERIALS AND METHODS

### Experimental design

The FEMTOPOWER V laser system (Spectra-Physics, Austria) delivers ultrashort laser pulses with a duration of 25 fs, operating at a repetition rate of 1 kHz with about 5 mJ of pulse energy at a central wavelength of 800 nm. The shortest pulse duration, measured just after the laser amplifier unit through autocorrelation at the optimal compressor grating pair position, is ~25 fs. Longer pulses, up to 100 fs, are generated by adjusting the separation between the compressor grating pair, with their durations estimated using the dispersion curve provided by the manufacturer. For pulses exceeding 75 fs, the pulse width is primarily determined by the grating compressor, with a residual error of about 10% (*48*). A reference experiment with opposite sign of the chirp was used to confirm no significant change in count rates or fragmentation patterns between long pulses of positive or negative dispersion similar to the one discussed in (*48*).

A λ/2 (B. Halle Nachfl. GmbH) plate and thin-film polarizer (Spectra-Physics, Austria) were used to control the laser power for the experiment. The laser power was continuously monitored with a power meter placed behind the output window of the mass spectrometer. The circular polarization of the beam was achieved by introducing an achromatic broadband quarter-wave plate (B. Halle Nachfl. GmbH). The Gaussian beam was converted into Laguerre-Gaussian beam using spiral phase plates (Holo/Or Ltd., Israel) which generate ℓ=±1 and ℓ=±2 for 800-nm light. Opposite topological charges of the OAM are generated by rotating the plate about its axis by 180°. The OAM and its charge of the output beam were independently measured by imaging its profile (figs. S4 and S5) using a single cylindrical lens and a camera, which is consistent with (*49*). A camera imaging the focal position within the spectrometer also ensured that the singularity of the generated ℓ=±1,±2 beam was always in the center (fig. S8).

The quarter-wave plate and spiral phase plate were mounted on rotation stages with a stepper motor. The programmed motor changed the handedness/polarization of the beam every 300 s. Thus, for a given intensity and pulse duration of the laser, mass spectra for different OAM/SAM combinations are obtained in the same run, mitigating effects due to long-term drifts.

A lens of *f* = 30 cm focuses the laser beam to a measured spot size of full width at half maximum radius of ~75 μm, leading to peak intensities of the order of 10^12^ to 10^13^ W/cm^2^. An ion velocity map imaging spectrometer equipped with a microchannel plate was used to detect the ions from the ionization region, run solely as a mass spectrometer. The ions originating from the source region are guided toward the detector via three electrodes and a drift tube as discussed in (*50*). Each experiment run, corresponding to different laser conditions, was performed for about 2 hours, with ion count rates of around 100 Hz.

(1S)-(−)- and (1R)-(+)-Camphor enantiomers, with purities of 99 and 98%, respectively, were used in the experiment and purchased from Sigma-Aldrich. Camphor was introduced into the ionization chamber with a residual gas pressure of 10^−8^ mbar by heating the sample crucible up to 60°C. A different crucible and gas line for each enantiomer was used to avoid contamination. Mass spectrometry was used between the experiments to confirm the absence of residual camphor. With the effusive gas jet on, the pressure in the chamber is maintained at ~3 × 10^−7^ mbar, with the help of an ultrahigh vacuum all-metal gas dosing valve.

All mass spectra presented in this work are normalized to the total ion yield of the corresponding spectrum. The experimental baseline is established using oxygen as an achiral reference system. The complete experimental protocol was repeated under identical conditions. As no intrinsic asymmetry is expected for an achiral system, the residual asymmetry measured for oxygen is interpreted as a direct measure of systematic contributions. The value of G for each fragment is obtained by taking the total normalized counts under each m/q peak.

### Theory

We begin by formulating the interaction between a chiral molecule and light carrying OAM within the Power-Zienau-Woolley Hamiltonian framework as (*51*)

$$H_{\text{int}}=-\sum_{\xi }\left[d_{i}\left(\xi \right)E_{i}^{⊥}\left(R_{\xi }\right)+q_{\text{ij}}\left(\xi \right)\nabla_{j}E_{i}^{⊥}\left(R_{\xi }\right)\right]$$ (2)

where *d*_i_ and *q*_ij_ represent the contributions of the electric dipole (E1) and the electric quadrupole (E2) interactions, respectively. $E_{i}^{⊥}$ is the transverse electric field component and $R_{\xi }$ is the coordinate of charge ξ. Crucially, the lowest-order interaction that enables chiral sensitivity to OAM light arises from interference between E1 and E2 transitions. This stems from the fact that E1 term is parity-odd, while E2 term is parity-even, thereby satisfying the necessity for chiral sensitivity (*18*). Note that the standard CD techniques exploit the interference between E1 and parity-even magnetic dipole (M1) transitions. To obtain the total photoinduced current, we need to evaluate the transition amplitude between the initial and final states. The total photoelectron current density, considering all laser-driven fragmentation momenta for a fixed molecular orientation, can be written as $j=\int d\Omega_{k}\;k\;{|a_{k}|}^{2}$, where $a_{k}$ is the transition amplitude and ***k*** is the momentum of the photoelectron (*52*). The transition amplitude for the Hamiltonian given in Eq. 2 can be expressed as

$$a_{k}=-\left\langle \psi_{k}|[d_{i}+q_{\text{ij}}]|\psi_{i}\right\rangle \left\langle (n-1)|[E_{i}^{⊥}\left(r\right)+\nabla_{j}E_{i}^{⊥}\left(r\right)]|n\right\rangle$$ (3)

Here, $|\psi_{i}\rangle$ and $|\psi_{k}\rangle$ are the initial and final states of the molecular electron, respectively. ∣n⟩ stands for the radiation state, which belongs to a specific mode of the Laguerre-Gaussian (LG) beam. Using the transverse electric field expansion of the LG beam (*18*), the above expression of the transition amplitude reads

$$a_{k}=-i{\left(\frac{\mathrm{n\hbar \omega}}{2\epsilon_{0}V}\right)}^{\frac{1}{2}}\varepsilon_{i}\left[D_{i}+Q_{\text{ij}}\nabla_{j}\right]f_{\ell ,p}\left(r\right)e^{i\left(\kappa z+\ell \varphi \right)}$$ (4)

Here, $\varepsilon_{i}$ is the polarization vector, n is the number of photons in volume V, κ is the wave vector of the LG beam, and $f_{\ell ,p}\left(r\right)$ is the appropriately normalized radial distribution of the LG beam with ℓ as the topological charge and p as the radial index. $D_{i}=\langle \psi_{k}|d_{i}|\psi_{i}\rangle$ and $Q_{\text{ij}}=\langle \psi_{k}|q_{\text{ij}}|\psi_{i}\rangle$ are E1 and E2 matrix amplitudes, respectively.

The key quantity in the total current density if the square of the transition amplitude, ${|a_{k}|}^{2}$ in which let us focus on the OAM-dependent terms as

$$\begin{array}{c} {|a_{k}|}^{2}=\\ \left(\frac{\text{nℏω}}{2\varepsilon_{0}V}\right){|f_{\ell ,p}\left(r\right)|}^{2}\left[\begin{array}{c} {|\hat{\varepsilon }\cdot \left(D-r^{-1}T\right)|}^{2}+{|(\ell r^{-1}\hat{\varepsilon }\cdot P)|}^{2}\\ +\left\{{\hat{\varepsilon }}^{*}\cdot \left(D^{*}-r^{-1}T^{*}\right)-\left(i\ell r^{-1}{\hat{\varepsilon }}^{*}\cdot P^{*}\right)\right\}\;\left\{\hat{\varepsilon }\cdot \left(D-r^{-1}T\right)+\left(i\ell r^{-1}\hat{\varepsilon }\cdot P\right)\right\} \end{array}\right]\\ . \end{array}$$ (5)

For simplification, we have considered $P_{i}=Q_{\text{ij}}{\hat{\phi }}_{j}$ and $T_{i}=Q_{\text{ij}}{\hat{r}}_{j}$. The cross terms, i.e., the terms appearing in the last line of the above equation, survive if and only if the polarization vector is imaginary. To simplify the presentation, we separate the polarization-dependent terms from the remaining contributions in the next step.

The total signal, corresponding to the photoelectron current density associated with the fragmentation process, reads

$$I_{\ell }\propto \int_{d\Omega_{k}}\left[\begin{array}{c} \left\{\left(D^{*}-r^{-1}T^{*}\right)\times \left(D-r^{-1}T\right).k\right\}+\left\{\left(\ell r^{-1}P^{*}\right)\times \left(\ell r^{-1}P\right)\right\}\cdot k\\ +\left\{\left(D^{*}-r^{-1}T^{*}\right)\times \left(\ell r^{-1}P\right)-\left(\ell r^{-1}P^{*}\right)\times \left(D-r^{-1}T\right)\right\}.k \end{array}\right]\left({\hat{\varepsilon }}^{*}\times \hat{\varepsilon }\right)$$ (6)

We have incorporated the expression for the OAM light beam to deduce the above expression.

The differential absorption of OAM light with *ℓ* > 0 and *ℓ* < 0 is obtained from the difference between $I_{\ell >0}$ and $I_{\ell <0}$, which is equivalent to helical dichroism in chiral molecules. After straightforward algebra, we obtain the expression for the helical dichroism, which is proportional to the contrast and can be written as

$$\begin{array}{c} \Sigma =I_{\ell >0}-I_{\ell <0}\\ =|\ell |\int_{d\Omega_{k}}\left[\left\{\left(D^{*}-r^{-1}T^{*}\right)\times \left(r^{-1}P\right)-\left(r^{-1}P^{*}\right)\times \left(D-r^{-1}T\right)\right\}\cdot k\right]\left({\hat{\varepsilon }}^{*}\times \hat{\varepsilon }\right) \end{array}$$ (7)

The above expression is nonzero only when the OAM beam carries SAM, i.e., for circularly polarized light. In contrast, for a linearly polarized OAM beam, the signal vanishes since the vector product $\left({\hat{\varepsilon }}^{*}\times \;\hat{\varepsilon }\right)$ becomes zero. This highlights the cooperative role of SAM and OAM in enhancing chiral sensitivity. Furthermore, the theoretical contrast exhibits a linear dependence on *ℓ*, which is in agreement with previous findings (*30*, *31*).
