# Supplementary material for: Enhanced chiral discrimination in mass spectrometry with orbital angular momentum beams
Source: Sci Adv. 2026 Jun 5;12(23):eaec6549. doi: 10.1126/sciadv.aec6549 (PMC13240229; doi:10.1126/sciadv.aec6549)
Supplement: Supplementary file 1 — Supplementary Text Figs. S1 to S8 Table S1 [file sciadv.aec6549_sm.pdf]

Science Advances
AAAS

Supplementary Materials for

Enhanced chiral discrimination in mass spectrometry with orbital angular momentum beams

Haritha Venugopal *et al.*

Corresponding author: Ram Gopal, ramgopal@tifrh.res.in; Vandana Sharma, vsharma@phy.iith.ac.in

Sci. Adv. **12**, eaec6549 (2026)
DOI: 10.1126/sciadv.aec6549

This PDF file includes:

Supplementary Text
Figs. S1 to S8
Table S1

Supplementary Text

Ellipticity Dependence of Contrast

To investigate the dependence of contrast on the polarization of the beam, measurements were performed at five polarization states: linear, left-circular (LCP), right-circular (RCP), and two intermediate ellipticities (one between LCP and linear, and the other between linear and RCP). The experiment was done with (1R)-(+)-Camphor at long pulse duration (850 fs). For each polarization state, the count rate was kept constant. The corresponding contrast values were obtained for each state, and the results are presented in Fig. S1 as a function of ellipticity. The measurements show a pronounced dependence of contrast on circular polarization. The contrast is highest with circularly polarized excitation (LCP and RCP) and reaches a minimum for linearly polarized light, with the intermediate points lying between these two limits.

Determination of the Stokes parameters

The polarization state of the laser beam used in the experiments was characterized by measuring its Stokes parameters to confirm the purity of linear and circular polarization states employed in the study. The measurements were performed using a standard polarization analysis setup consisting of a linear polarizer (analyzer) and a quarter-wave plate, followed by intensity detection with a power meter. The Stokes parameters (S_0, S_1, S_2, S_3) provide a complete description of the beam polarization, where S_0 denotes the total intensity, S_1 and S_2 represent the linear polarization components, and S_3 quantifies the degree of circular polarization. For linearly polarized input light, the analysis confirmed dominant linear polarization. For circularly polarized light, generated using the quarter-wave plate, the measurements yielded dominant S_3 components with minimal residual linear polarization, indicating high polarization purity. All Stokes parameters are normalized to the total intensity S_0 (Table S1).

Effect of Chirp on Measured Contrast

To assess the role of pulse chirp on the chiral contrast G, the pulse duration was tuned from the shortest attainable value of 25 fs to positively and negatively chirped values of +850 fs and -850 fs. Intermediate points at +450 fs and -450 fs were also included for comparison. Across this range, the addition of chirp did not introduce a marked disparity between positively and negatively chirped pulses; both yielded comparable values of G indicating that the magnitude of temporal

stretching, rather than its sign, governs the observed behavior, as shown in Fig S2 (a). Throughout these measurements, the ion rate was held constant to ensure that variations in pulse duration did not bias the excitation intensity, allowing a direct comparison of chirp-dependent effects. In Fig S2 (b) the pulse width dependence is plotted (not at the same laser parameters as in the chirp experiment).

Estimation of the Systematic Experimental Error Using an Achiral Reference

To establish the baseline error of the measurement scheme, control experiments were performed using molecular Oxygen O_2 as an achiral reference system. Owing to the absence of structural chirality, Oxygen is not expected to exhibit any genuine contrast. Consequently, any finite contrast observed under identical measurement conditions reflects residual instrumental contributions inherent to the setup. The Oxygen measurements were carried out under the same experimental conditions as the Camphor experiments, including identical optical, ion-optical, detection, and data analysis settings, ensuring direct comparability between the reference and chiral datasets. Measurements were first conducted using configuration $(\ell = 0, s = +1)$ and $(\ell' = 0, s' = -1)$ (CD) for which a contrast value of 0.05 was obtained. The experiment was subsequently repeated using configurations $(\ell = +1, s = 0)$ and $(\ell' = -1, s' = 0)$; and $(\ell = +1, s = +1)$ and $(\ell' = -1, s' = +1)$ yielding contrast values of 0.07 (Fig. S3) in both cases. Since these signals originate from an achiral molecule, they are attributed to non-physical asymmetries introduced by the measurement system. Based on these observations, the contrast *G* obtained for Oxygen molecule is adopted as the baseline error level of the setup.

Automation of Experiment

The measurements were carried out using an automated control scheme to maintain consistent experimental conditions during long acquisition times and to limit variations arising from manual operation. The purpose of the automation was to reduce cumulative errors associated with extended measurements. The spiral phase plate (SPP) and the quarter-wave plate (QWP) were mounted on separate motorized rotation stages and used to switch between predefined optical configurations. The SPP was mounted on a Newport piezo-driven rotation stage, while the QWP was mounted on a rotation stage driven by a stepper motor. Switching between configurations was performed at fixed intervals of 300 s. During each change of configuration, the laser beam was blocked to prevent data acquisition while the optical elements were in motion. Beam blocking was implemented using a servo-actuated shutter synchronized with the rotation stages, such that signals

were recorded only under stationary optical conditions. Stage motion, shutter control, and acquisition timing were coordinated using a control program written in C#.

Synchronization between the optical configuration and the detection electronics was achieved using a DS1023 delay generator, which converted the control voltage corresponding to the stage position into a programmable nanosecond-scale delay. This timing signal was provided to the time-to-digital converter (TDC), enabling each recorded event to be associated with the corresponding optical configuration. A TTL signal taken directly from the laser system was used as the trigger input to the DS1023. This arrangement provided reproducible switching of the beam handedness and reliable temporal tagging of the experimental configuration throughout the data acquisition.

Characterization of Orbital Angular Momentum

The orbital angular momentum (OAM) of the beam was characterized using a cylindrical lens with a focal length of 50 mm introduced into the beam path, and the transverse intensity distribution was recorded using a camera positioned at the focal plane of the lens. This procedure enables discrimination between opposite OAM signs through the characteristic rotation of the intensity pattern. Measurements were performed for $\ell = +1$ and $\ell = -1$ to verify the handedness switching implemented in the experiment (Fig. S4). In addition, reference images were acquired for $\ell = 0$ to confirm the expected mode structure. Measurements for $\ell = +2$ and $\ell = -2$ are shown in Fig. S5.

Effect of beam misalignment

The influence of misalignment on the measured asymmetry was examined by deliberately displacing the phase singularity from the optical axis. The singularity was shifted horizontally and vertically by approximately 13 μm and 24 μm, respectively, relative to the centered position. For each displacement, the asymmetry was evaluated by calculating the contrast between the signal obtained at the centered configuration and that for the shifted.

These measurements were performed for (1S)-(-)-Camphor using a long laser pulse duration (850 fs) and optical configuration of $(\ell = +1, s = +1)$. The resulting contrast values for different fragment ions, obtained for both horizontal and vertical displacements, are presented in Figures (Fig. S6 and Fig. S7) below.

To verify that the beam position remained unchanged after reversal of the handedness, the focal plane of the interaction region was imaged using a camera mounted at the exit window of the vacuum chamber. The recorded profiles (Fig. S8) showed that the beam returned to the same

spatial location after switching, indicating that the optical alignment was preserved throughout the handedness changes.

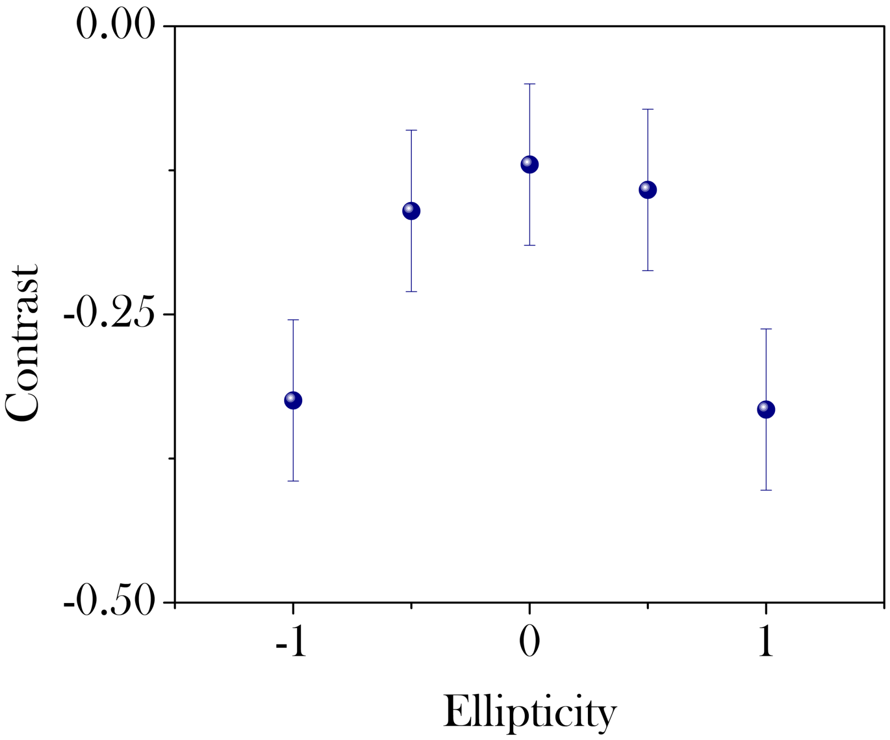

Fig. S1: Contrast of (1R)-(+)-Camphor as a function of ellipticity.

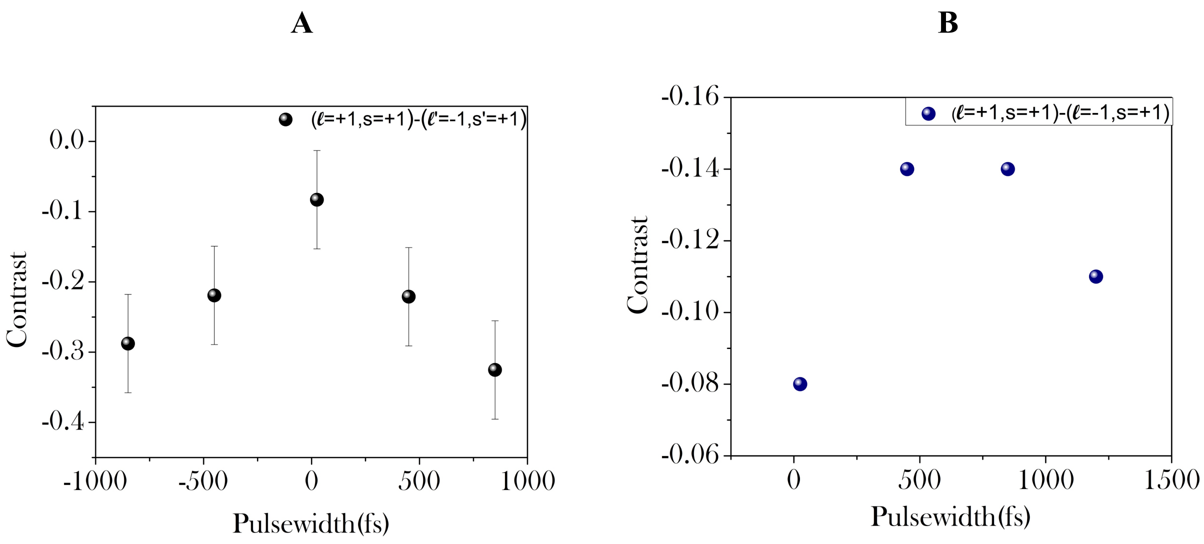

Fig. S2: Contrast of (1R)-(+)-Camphor as a function of (A) chirp and (B) pulse duration.

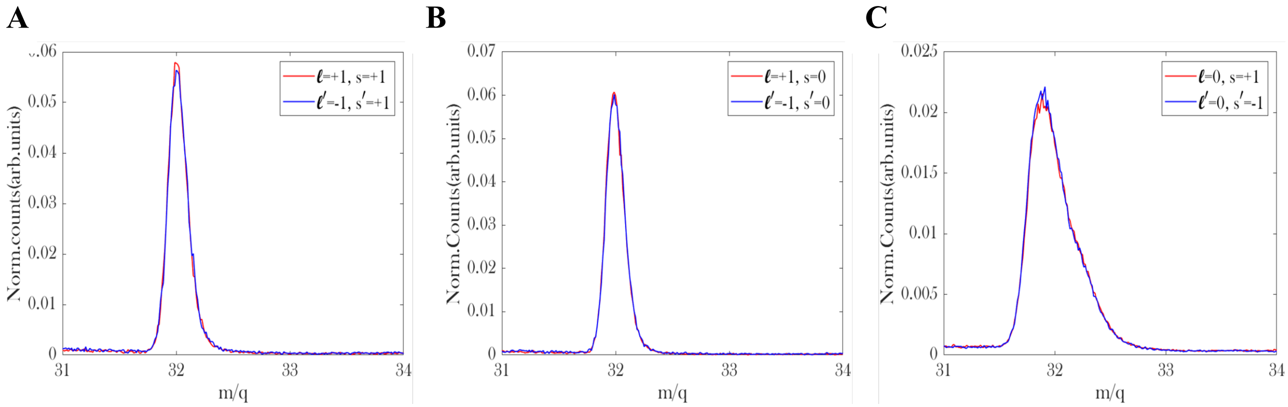

Fig. S3: Oxygen mass spectra recorded under three different SAM–OAM configurations. The associated contrast values for each case are presented in panels (**A**) 0.07, (**B**) 0.07, and (**C**) 0.05, respectively.

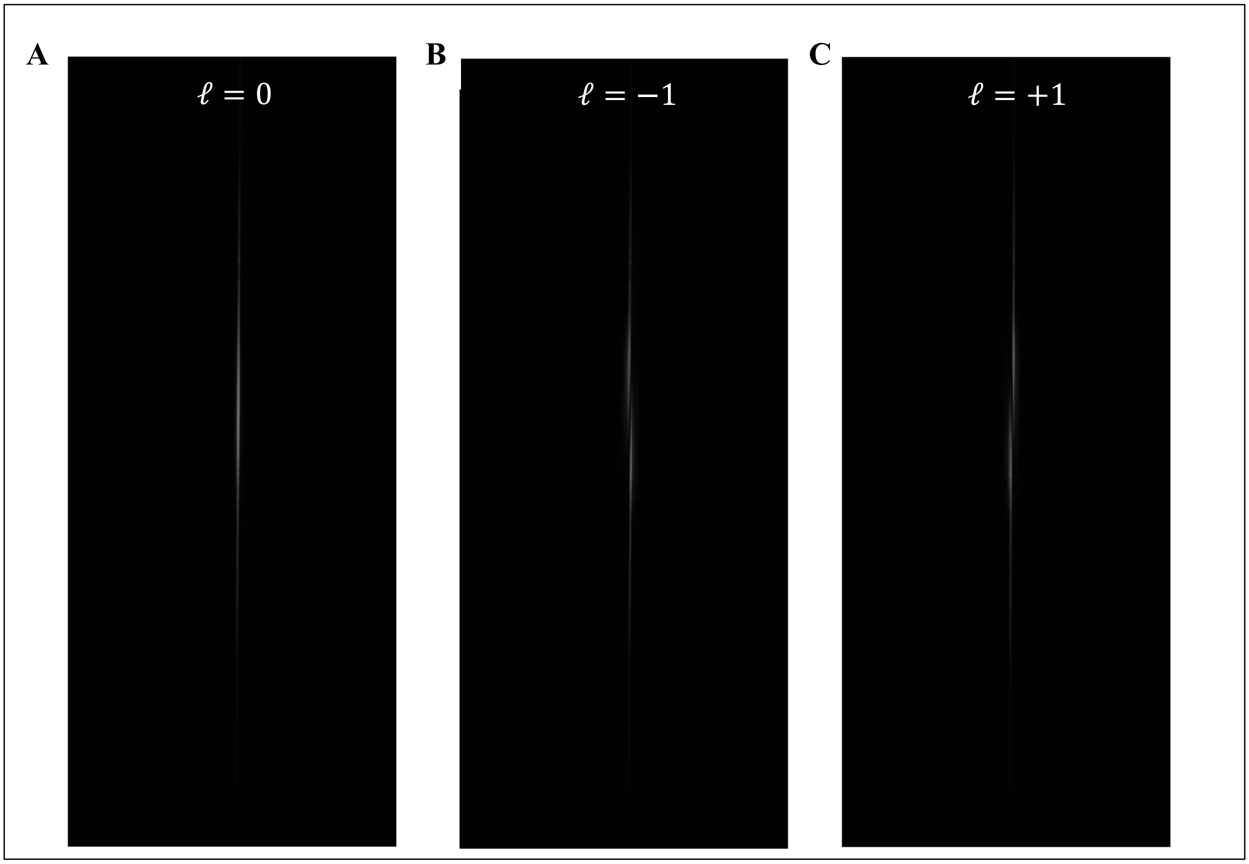

Fig. S4: Cylindrical lens output for sign determination of the OAM beam for (**A**) $\ell = 0$, (**B**) $\ell = -1$ and (**C**) $\ell = +1$.

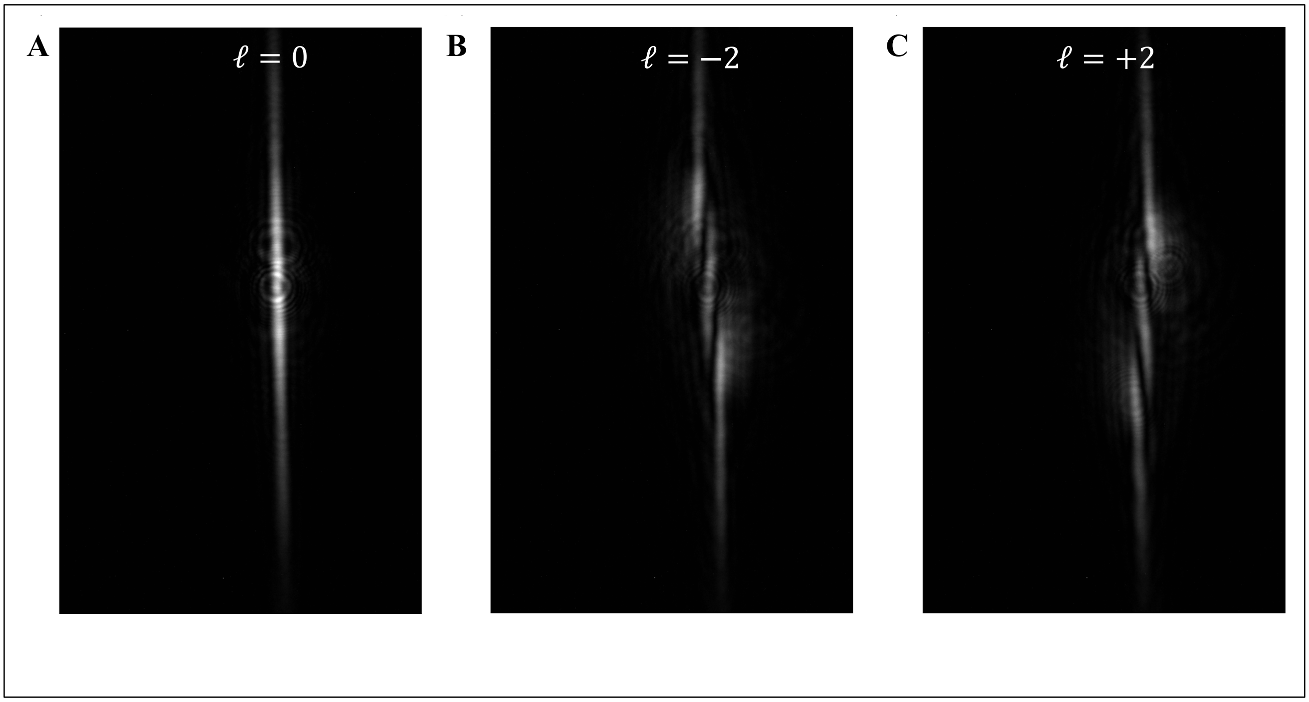

Fig. S5: Cylindrical lens output for sign determination of the OAM beam for (**A**) $\ell = 0$, (**B**) $\ell = -2$ and (**C**) $\ell = +2$.

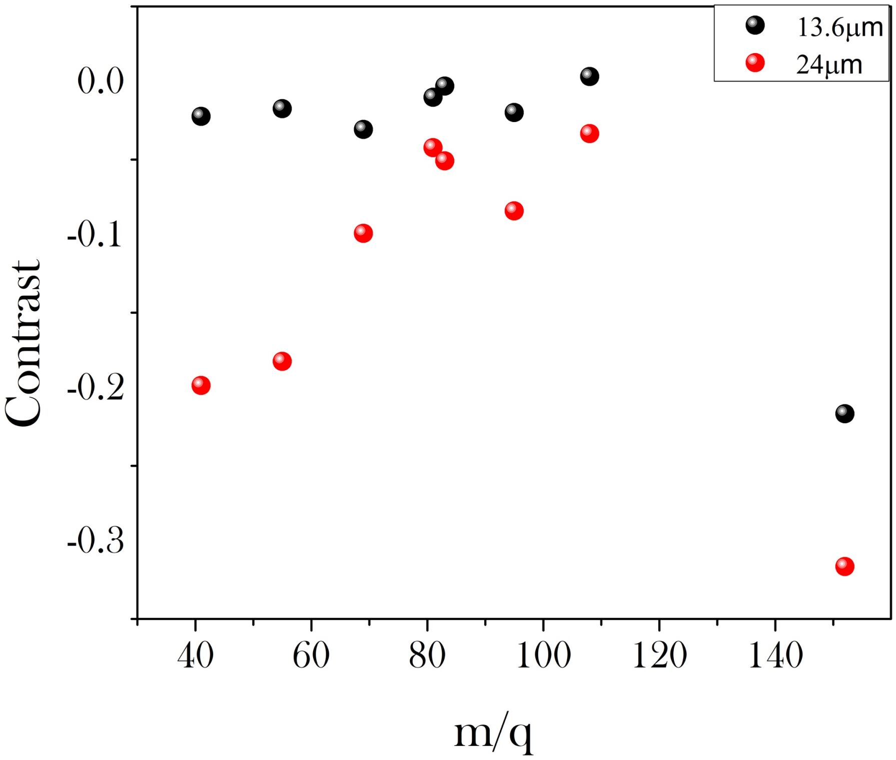

Fig. S6: The contrast of fragment ions for horizontal displacements of the singularity, from the beam center. The singularity was shifted sequentially to two off-axis positions located at 13.6 μm and 24.0 μm from the center.

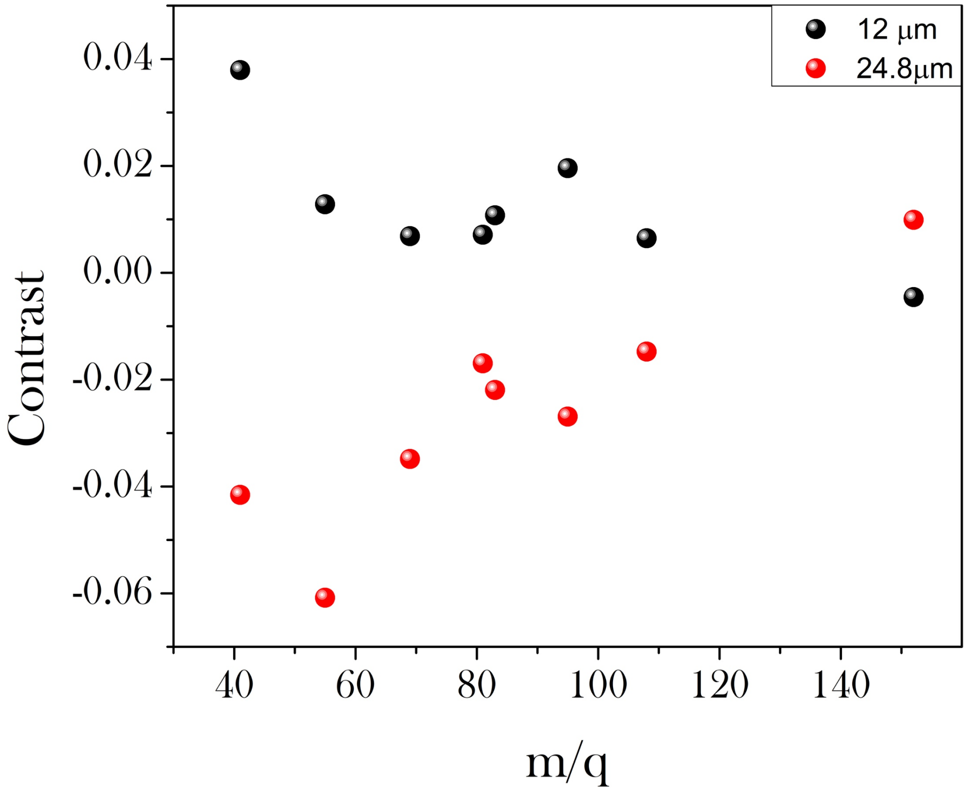

Fig. S7: The contrast of fragment ions for vertical displacements of the singularity, from the beam center. For each direction, the singularity was shifted sequentially to two off-axis positions located at 12.0 μm and 24.8 μm from the center.

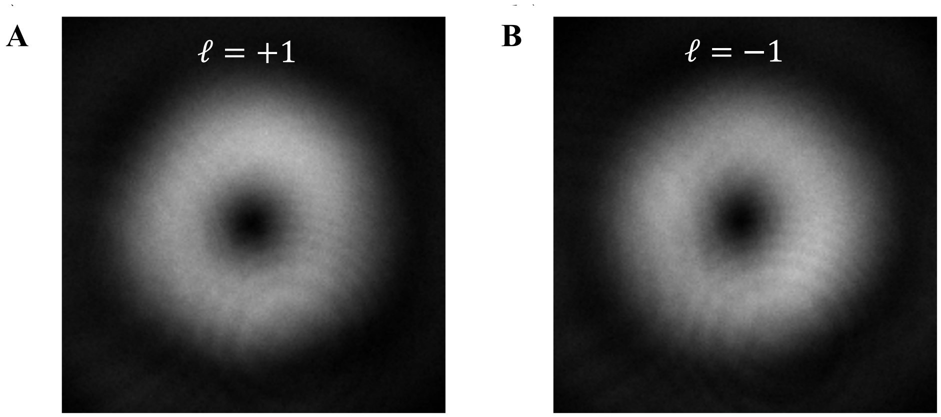

Fig. S8: Camera images recorded at the exit window showing the imaged focal spot for an optical vortex beam with topological charge (**A**) $\ell = +1$ and (**B**) $\ell = -1$.

Polarisation	S_0	S_1	S_2	S_3
Linear	1	0.92	-0.107	0.36
Circular	1	0.09	-0.04	0.94

Table S1. Measurement of Stokes parameters
